# Evaluating the risk of knee osteoarthritis following unilateral ACL reconstruction based on an EMG-assisted method

**DOI:** 10.3389/fphys.2023.1160261

**Published:** 2023-04-21

**Authors:** Ting Long, Justin Fernandez, Hui Liu, Hanjun Li

**Affiliations:** ^1^ Auckland Bioengineering Institute, University of Auckland, Auckland, New Zealand; ^2^ Biomechanics Laboratory, Beijing Sport University, Beijing, China; ^3^ Department of Engineering Science, University of Auckland, Auckland, New Zealand; ^4^ China Institute of Sport and Health Science, Beijing Sport University, Beijing, China

**Keywords:** anterior cruciate ligament reconstruction, knee osteoarthritis, tibial contact force, EMG-assisted model, gait

## Abstract

**Objective:** Anterior cruciate ligament reconstruction (ACLR) cannot decrease the risk of knee osteoarthritis after anterior cruciate ligament rupture, and tibial contact force is associated with the development of knee osteoarthritis. The purpose of this study was to compare the difference in bilateral tibial contact force for patients with unilateral ACLR during walking and jogging based on an EMG-assisted method in order to evaluate the risk of knee osteoarthritis following unilateral ACLR.

**Methods:** Seven unilateral ACLR patients participated in experiments. The 14-camera motion capture system, 3-Dimension force plate, and wireless EMG test system were used to collect the participants’ kinematics, kinetics, and EMG data during walking and jogging. A personalized neuromusculoskeletal model was established by combining scaling and calibration optimization. The inverse kinematics and inverse dynamics algorithms were used to calculate the joint angle and joint net moment. The EMG-assisted model was used to calculate the muscle force. On this basis, the contact force of the knee joint was analyzed, and the tibial contact force was obtained. The paired sample *t*-test was used to analyze the difference between the participants’ healthy and surgical sides of the participants.

**Results:** During jogging, the peak tibial compression force on the healthy side was higher than on the surgical side (*p* = 0.039). At the peak moment of tibial compression force, the muscle force of the rectus femoris (*p* = 0.035) and vastus medialis (*p* = 0.036) on the healthy side was significantly higher than that on the surgical side; the knee flexion (*p* = 0.042) and ankle dorsiflexion (*p* = 0.046) angle on the healthy side was higher than that on the surgical side. There was no significant difference in the first (*p* = 0.122) and second (*p* = 0.445) peak tibial compression forces during walking between the healthy and surgical sides.

**Conclusion:** Patients with unilateral ACLR showed smaller tibial compression force on the surgical side than on the healthy side during jogging. The main reason for this may be the insufficient exertion of the rectus femoris and vastus medialis.

## 1 Introduction

Anterior cruciate ligament (ACL) injury is one of the most common sports-related injuries (Agel et al., 2005; Yu and Garrett, 2007), and anterior cruciate ligament reconstruction (ACLR) cannot decrease the risk of knee osteoarthritis. A study suggested that patients with a single ACL rupture have a 10%–13% risk of developing knee osteoarthritis 10 years after injury (Oiestad et al., 2009). This risk increases from 21% to 48% in patients with a torn meniscus associated with ACL rupture (Oiestad et al., 2009). ACLR surgery is a common treatment for ACL rupture, but previous studies have shown that ACLR surgery cannot reduce the incidence of knee osteoarthritis (Lohmander et al., 2007; Barenius et al., 2014; Cinque et al., 2018; Grassi et al., 2022). A study showed that the incidence of knee osteoarthritis on the ACLR surgical side increased by three times compared with the contralateral side (Barenius et al., 2014). Studies have also shown that, on average, 50% of patients with ACL or meniscus tears will develop knee osteoarthritis after 10–20 years (Lohmander et al., 2007). Despite the high incidence of knee osteoarthritis after ACLR, the exact cause remains unclear.

There will be a series of changes in biomechanical characteristics for ACLR patients in daily activities, which may increase the risk of knee osteoarthritis. Studies conducted on patients who have undergone ACLR have reported a decrease in peak knee flexion angles (Roewer et al., 2011; Hall et al., 2012; Webster et al., 2012; Saxby et al., 2016), knee flexion moments (Bush-Joseph et al., 2001; Lewek et al., 2002; Webster et al., 2005; Di Stasi et al., 2013; Zabala et al., 2013), and external knee adduction moments (Webster and Feller, 2012; Zabala et al., 2013; Kaur et al., 2016) during prolonged walking or jogging after the surgery. Additionally, ACLR patients’ muscle activation strategy also changed at the same time, which is known in rehabilitation field as arthrogenic muscle inhibition. After ACLR, quadriceps activation failure caused by neural inhibition occurs (Sonnery-Cottet et al., 2019). Mechanisms for this phenomenon include changes in muscle resting motor thresholds, in the discharge of articular sensory receptors (Rice and McNair, 2010) and in the cortical activity (Baumeister et al., 2008; Baumeister et al., 2011). Arthrogenic muscle inhibition also shows in the daily movement. Several studies’ results suggested that there is an increase in hamstring activation (Knoll et al., 2004; Mantashloo et al., 2020) and a decrease in quadriceps femoris activation (Leporace et al., 2016) among ACLR patients during walking. The altered biomechanics in movement will significantly affect the mechanical environment of the knee joint, which may increase the risk of knee osteoarthritis. Bone loads are crucial to the growth and remodeling process of joint tissues (Beaupre et al., 2000; Carter et al., 2004). There is a dynamic balance between the reabsorption of bone matrix by osteoclasts and the formation of new bone by osteoblasts. However, this dynamic balance can be disrupted by abnormal loads, which may cause degenerative changes in joint tissue (Radin and Paul, 1971). Therefore, assessing tibial contact forces can improve understanding of knee osteoarthritis mechanisms.

The contribution of muscle forces cannot be ignored when assessing the tibial contact force, but evaluating muscle forces non-invasively during movement has always been a challenge because the human musculoskeletal system has redundancy (Crowninshield and Brand, 1981; Lloyd and Besier, 2003). Two studies estimated tibial contact forces for ACLR patients, and both used the EMG-driven method to evaluate muscle forces during a gait task (Saxby et al., 2016; Wellsandt et al., 2016). Wellsandt et al. (Wellsandt et al., 2016) suggested that patients who get knee osteoarthritis 5 years after ACLR surgery have a smaller tibial compression force during walking. Saxby et al. (Saxby et al., 2016) obtained the consistent result that the patients in the ACLR group show a smaller tibial compression force than the healthy group during walking, jogging, and cutting. Although the EMG-driven method uses EMG signal as input to the musculoskeletal simulation to solve the muscle redundancy problem, it still has some limitations like the difficult measurement of EMG signal of deep muscles and the large error with experimental results caused by signal noise (Sartori et al., 2014; Hoang et al., 2019). The EMG-assisted method combines the optimization and EMG-driven methods, which can significantly improve the experimental joint moment tracking accuracy than current EMG-driven models (Sartori et al., 2014; Pizzolato et al., 2015; Hoang et al., 2019).

The purpose of this study was to compare the difference in bilateral tibial contact force for patients with unilateral ACLR during walking and jogging based on an EMG-assisted method to evaluate the risk of knee osteoarthritis. We hypothesized that: 1) ACLR patients’ tibial compression force on the surgical side would be smaller than on the healthy side during walking and jogging; 2) there would be difference in muscle forces around the knee joint between the surgical and healthy sides during walking and jogging; 3) there would be difference in lower limb kinematics between the surgical and healthy sides during walking and jogging.

## 2 Materials and methods

### 2.1 Participants

Seven ACLR participants (three males and four females, height: 1.72 ± 0.13 m, weight: 67.4 ± 14.3 kg, 1.5–3.5 years post-ACLR, the surgical side of the five participants was the dominant side) participated in this study. None of the participants had other lower limb injuries and did not engage in high-intensity exercise within 24 h before the test. The study protocol and ethics were approved by the Institutional Review Board of Beijing Sport University (2019018H).

### 2.2 Procedures

Before the gait test, the participant was in tight-fitting clothes and self-provided running shoes. Then participants warmed up and practiced to become familiar with the test process and ensure that they could step on the force plate naturally and accurately during the formal test. After that, a total of 23 reflective markers were placed on the participant’s vertex of the head, the upper edge of the sternum, the midpoint of the fourth and fifth lumbar vertebrae, and bilaterally on acromions, anterior superior iliac spines, anterior thighs, medial femoral condyles, lateral femoral condyles, tibial trochanters, medial tibial condyles, lateral fibular condyles, heels, and toe tips. Surface electrodes were placed bilaterally on the participant’s long head of biceps femoris, semitendinosus, rectus femoris, vastus medialis, vastus lateralis, medial head of gastrocnemius, and lateral head of the gastrocnemius. The skin was shaved and exfoliated, then cleaned with alcohol before electrode placement.

In the gait test, participants performed repeated trials twice for each movement, including walking and jogging at their self-selected speed on both sides. During the test, participants started from 10 m away from the force plate and walked or jogged through the data collection area at their self-selected speed. During the whole process, participants kept smooth movements without pace adjustment. The action cycle of walking was defined from the tested foot strike and untested foot off to the tested foot strike again. The action cycle of jogging was defined from the tested foot strike to the tested foot strike again.

Muscle maximum voluntary contractions (MVCs) tests were performed after the gait test. During MVC tests of the medial head of gastrocnemius and lateral head of gastrocnemius, the participants were held in a standing position with their shoulders immobilized and used maximal effort to raise their heels. During MVC tests of the rectus femoris, vastus medialis, and vastus lateralis, participants were in a seated position with knee flexion angles of about 90 degrees. After fixing the participants’ shank and thigh, they used maximal effort to extend the knee. During MVC tests of the long head of biceps femoris and semitendinosus, participants were in a seated position with knee flexion angles of about 90 degrees. After fixing the participants’ shank and thigh, they used maximal effort to bend the knee. MVCs tests were repeated three times (5 s each) on both sides for each participant, with 1-min rest between repetitions.

### 2.3 Data collection

The reflective marker trajectories in the gait test were captured using a 14-camera motion capture system (Qualisys, Gothenburg, Sweden) at 200 Hz. The ground reaction force (GRF) in the gait test was obtained using three 3-D force plates (Kistler Instruments, Winterthur, Switzerland) at 1000 Hz. Electromyographic (EMG) data were recorded using a wireless test system (Delsys Trigno Mobile, USA) at 2000 Hz. The acquisition of marker trajectories, GRF, and EMG data were synchronized by signals from Qualisys external triggers.

### 2.4 Data processing and analysis

Marker trajectories, GRF, and EMG data were processed by an open-source Matlab (The Mathworks, MA) toolbox (MOtoNMS v2.2). GRF data were filtered using a second-order Butterworth low-pass filter at 50 Hz. The marker trajectories data were filtered using a second-order Butterworth low-pass filter at 13 Hz. The raw EMG data were removed mean, bandpass-filtered (second-order Butterworth, 50–450 Hz), full-wave rectified, and low-pass filtered (second-order Butterworth, 6 Hz) to produce linear envelopes. EMG envelopes were standardized using the maximum value during gait and MVCs tests.

To obtain a participant’s personalized model, a generic musculoskeletal model (gait 2,392) was scaled in OpenSim version 4.0 (Stanford, CA, United States). The scaling setting detail was in the Supplementary Material (Supplementary Table S1). The 3-D marker trajectories and GRFs collected in the gait test were used to calculate joint angles and moments through the Inverse Kinematics and Inverse Dynamics (ID) tools in OpenSim. Muscle analysis tools in OpenSim were used to calculate muscle-tendon unit (MTU) lengths and moment arms.

Muscle forces were estimated using an EMG-assisted method in the OpenSim plug-in CEINMS (Pizzolato et al., 2015). Based on the purpose of this study, we only analyzed the muscle forces around knee joints. There were two stages for muscle force estimation. In the first stage, data from one of the two repeated trials were used to calibrate the neuromuscular parameters of the personalized model. Seven experimental muscle excitations were mapped to 12 MTUs based on previous studies (Lloyd and Besier, 2003; Sartori et al., 2014; Hoang et al., 2019) (Supplementary Table S2). Then, the calibration process was performed in CEINMS to adjust the neuromuscular parameters to minimize joint moment prediction errors for the knee flexion-extension. The neuromuscular parameters included tendon slack length, optimal fiber length, maximum isometric force, non-linear shape factor, and EMG-to-activation recursive filter coefficients (Lloyd and Besier, 2003). The default value of neuromuscular parameters was from the scaled model. In the second stage, the calibrated neuromuscular model was used to predict muscle forces through the EMG-assisted model in CEINMS in the remaining trials. The objective function was:

$$F_{obj}=E_{trackMOM}+E_{trackEMG}+E_{sumEMG}$$

where E_trackMOM_ is the sum of the squared differences between EMG-assisted predicted and experimental joint moments, E_trackEMG_ is the sum of the squared differences between EMG-assisted predicted and experimental muscle excitations for seven MTUs recorded in the gait test, E_sumEMG_ is the sum of squared muscle excitations for all 12 MTUs. After predicting muscle forces, tibial contact forces (knee joint applied to tibia segment) were calculated using the Joint Reaction Analysis tools in OpenSim. The reference coordinate system was the tibia coordinate system. Results of muscle forces, joint moments, and tibial contact forces were normalized to body mass.

The muscle co-contraction index (CCI) was used to reflect muscle activation strategy (Hoang et al., 2019). We analyzed the CCI of knee flexors-extensors (CCI_flex-ext_) in this study. The calculating function was:

$${CCI}_{flex-ext}=\left\{\begin{array}{cc} 1-\frac{M\left(ext\right)}{M\left(flex\right)}, & M\left(ext\right)<M\left(flex\right)\\ \frac{M\left(flex\right)}{M\left(ext\right)}-1, & otherwise \end{array}\right.$$

where M (ext) is the mean value of knee extensors activation (rectus femoris, vastus medialis, and vastus lateralis, vastus intermedius); M (flex) is the mean value of major knee flexors activation (long head of biceps femoris, semitendinosus, semimembranosus, medial head of gastrocnemius, lateral head of the gastrocnemius). When CCI_flex-ext_ = 0, means full co-contraction; when CCI_flex-ext_ = 1, means no co-contraction and only flexor activation; when CCI_flex-ext_ = −1, means no co-contraction and only extensor activation.

Tibial compression forces calculated by the musculoskeletal were qualitatively compared with available instrumented knee implant data in the OrthoLoad dataset (Bergmann and Graichen, 2008; Bergmann et al., 2014) (Jog: a 70-year-old male, weighing 81.5 kg and standing 1.74 m, who jogged at a constant speed of 1.67 m/s on a treadmill; Walk: a 74-year-old female weighing 69.1 kg and standing 1.66 m, who walked at a speed of 1.11 m/s on a treadmill with bare feet) (Supplementary Figure S1). The coefficient of determination (R^2^) and root-mean-square error (RMSE) was calculated for the EMG-assisted predicted and ID knee flexion-extension net moment, which was used to evaluate EMG-assisted prediction results. Shapiro-Wilk test was used to determine the normality of lower limb biomechanical indexes on both sides. The paired sample *t*-test was used to analyze the difference in lower limb biomechanics between the participants’ healthy and surgical sides. A type I error rate no greater than 0.05 was chosen as an indication of statistical significance. All statistical analyses were performed using SPSS 22.0 software (SPSS, Chicago, IL, United States).

## 3 Results

**FIGURE 1:**
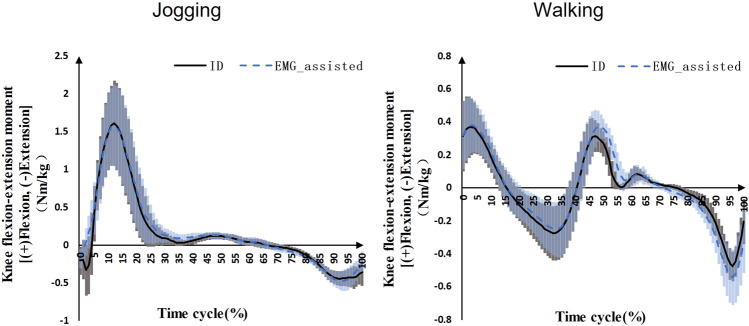
Knee flexion-extension net moments during jogging and walking (Black solid line: mean value of ID results on the both sides for seven patients, blue dashed line: mean value of EMG-assisted results on the both sides for seven patients, grey area: standard deviation of ID results on the both sides for seven patients, blue area: standard deviation of EMG-assisted results on the both sides for seven patients).

The knee flexion-extension net moment predicted by the EMG-assisted method showed high R^2^ and low RMSE with ID results during jogging (R^2^ = 0.95 ± 0.05, RMSE = 0.11 ± 0.05 Nm/kg) and walking (R^2^ = 0.94 ± 0.04, RMSE = 0.06 ± 0.03 Nm/kg) (Figure 1).

Participants’ action cycle on the healthy side was significantly longer than the surgical side during jogging (healthy = 0.741 ± 0.031 s, surgical = 0.721 ± 0.033 s, *p* = 0.009) and walking (healthy = 0.925 ± 0.023 s, surgical = 0.909 ± 0.026 s, *p* = 0.014) (Table 1). Participants’ stance phase in jogging (healthy = 0.256 ± 0.013 s, surgical = 0.234 ± 0.017 s, *p* = 0.005) and swing phase in walking (healthy = 0.256 ± 0.013 s, surgical = 0.234 ± 0.017 s, *p* = 0.005) on the healthy side was significantly longer than the surgical side (Table 1). There was no difference in other characteristics.

**TABLE 1:** Basic gait characteristics in jogging and walking.

	Jog	Jog	*p*-value	Walk	Walk	*p*-value
	Healthy side	Surgical side		Healthy side	Surgical side	
Step length (cm)	94.31 ± 5.53	95.76 ± 3.04	0.279	85.40 ± 3.09	82.72 ± 5.41	0.125
Velocity (m/s)	2.49 ± 0.13	2.54 ± 0.10	0.172	1.57 ± 0.06	1.62 ± 0.09	0.081
Action cycle (s)	0.741 ± 0.031	0.721 ± 0.033^*^	**0.009**	0.925 ± 0.023	0.909 ± 0.026^*^	**0.014**
Stance phase (s)	0.256 ± 0.013	0.234 ± 0.017^*^	**0.005**	0.485 ± 0.017	0.486 ± 0.024	0.411
Swing phase (s)	0.485 ± 0.040	0.487 ± 0.038	0.351	0.440 ± 0.010	0.422 ± 0.006^*^	**0.002**

*Significant difference between groups (*p* ≤ 0.05).

The bold values were used to highlight the *p*-values ≤ 0.05.

**FIGURE 2:**
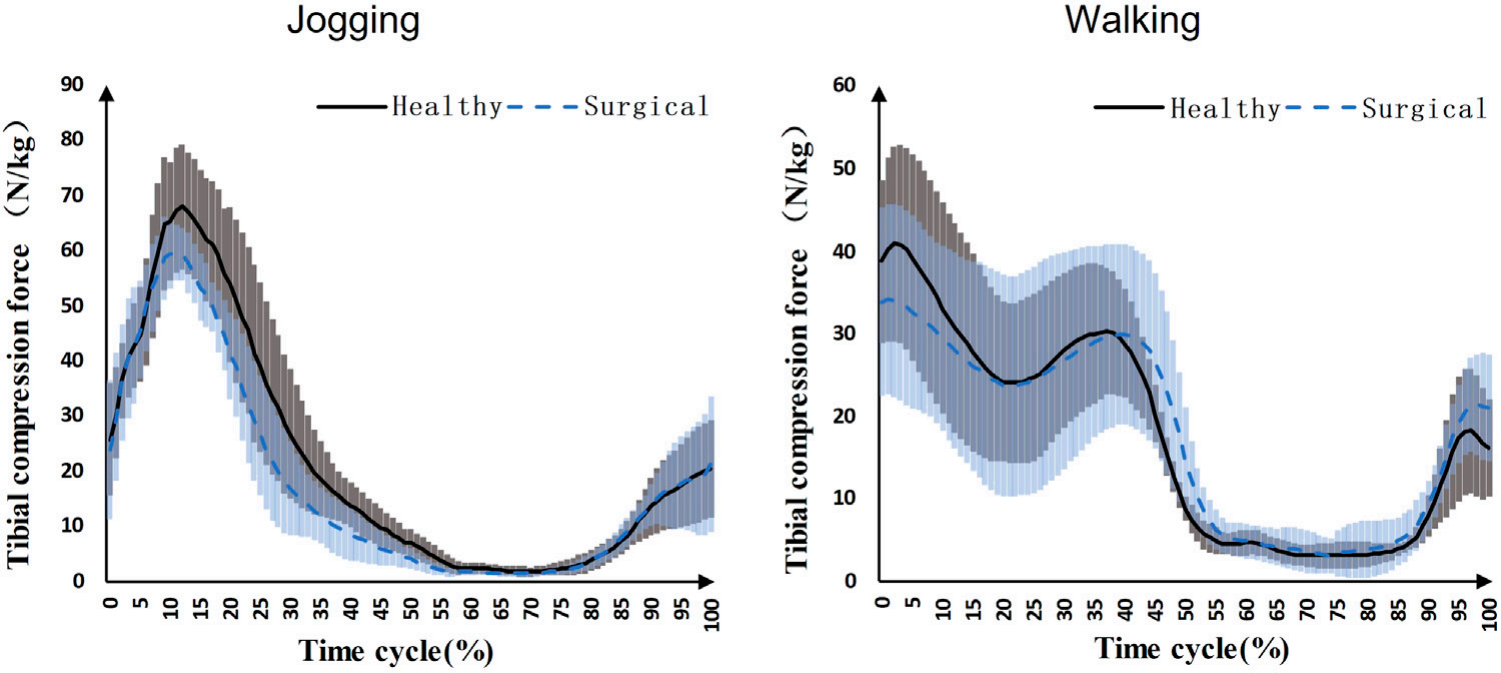
Knee compression forces on both sides during jogging and walking (Black solid line: mean value of results on the healthy sides for seven patients, blue dashed line: mean value of results on the surgical sides for seven patients, grey area: standard deviation of results on the healthy sides for seven patients, blue area: standard deviation of results on the surgical sides for seven patients).

Participants’ peak tibial compression force on the healthy side (68.76 ± 11.95 N/kg) was significantly higher than the surgical side (61.86 ± 5.46 N/kg) during jogging (*p* = 0.039) (Figure 2). There was no significant difference in the first (healthy = 43.00 ± 12.74 N/kg, surgical = 35.05 ± 12.61 N/kg, *p* = 0.122) and second (healthy = 31.12 ± 8.96 N/kg, surgical = 31.72 ± 14.32 N/kg, *p* = 0.445) peak tibial compression forces during walking between the healthy and surgical side (Figure 2).

**FIGURE 3:**
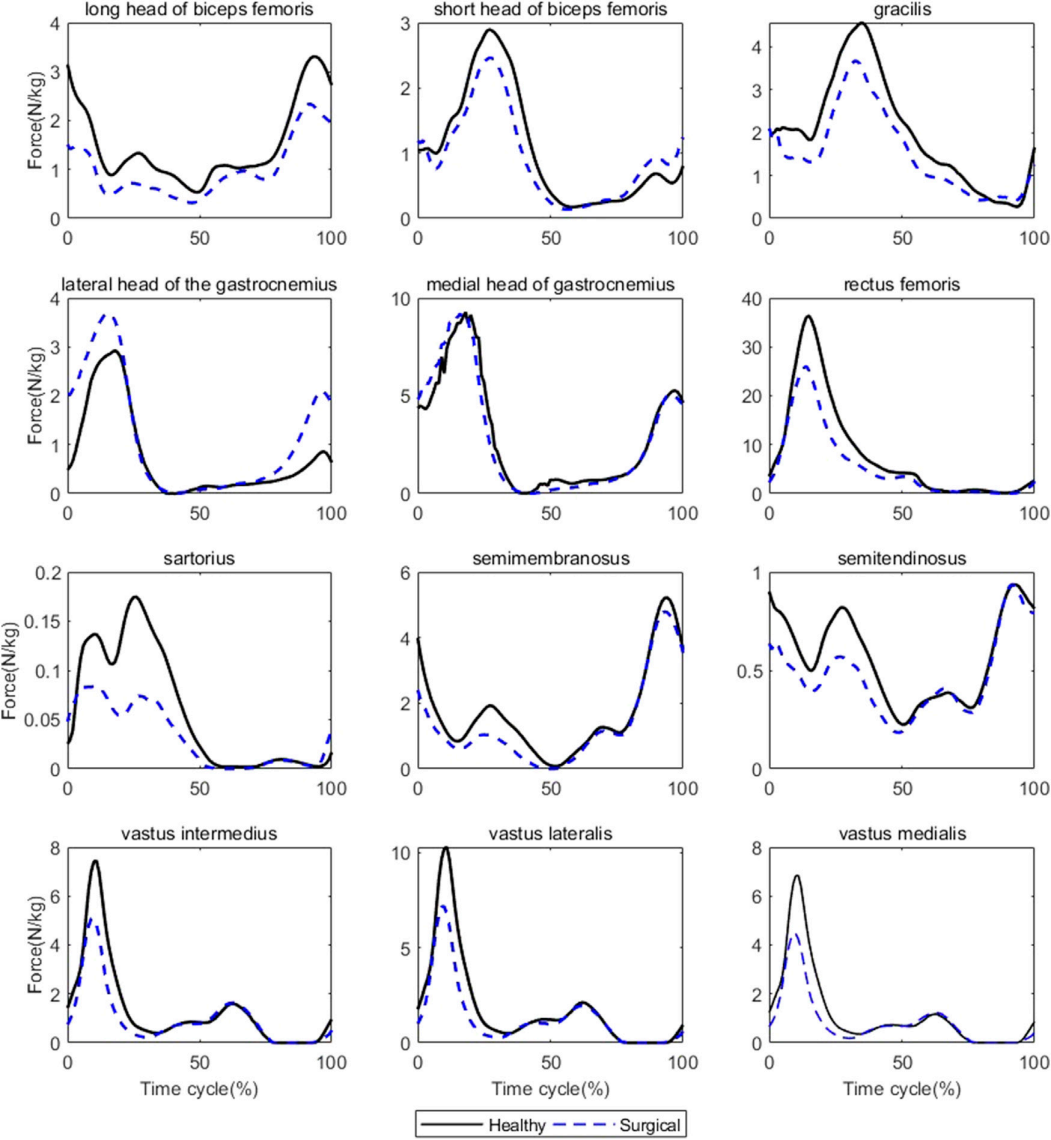
Muscle forces on both sides during jogging (Black solid line: mean value of results on the healthy sides for seven patients, blue dashed line: mean value of results on the surgical sides for seven patients).

**FIGURE 4:**
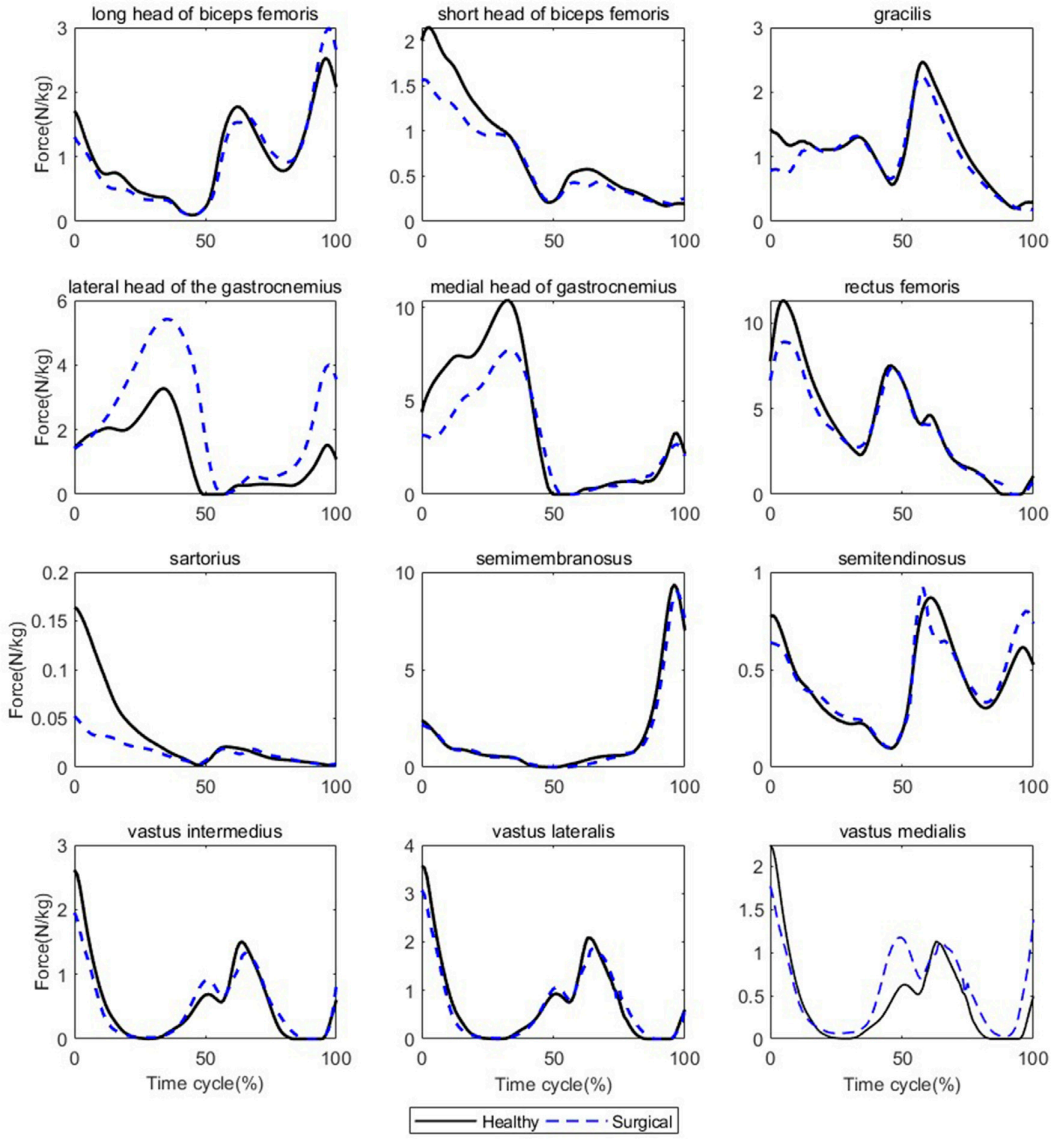
Muscle forces on both sides during walking (Black solid line: mean value of results on the healthy sides for seven patients, blue dashed line: mean value of results on the surgical sides for seven patients).

Participants’ rectus femoris (healthy = 32.08 ± 10.79 N/kg, surgical = 22.58 ± 9.16 N/kg, *p* = 0.035) and vastus medialis (healthy = 6.08 ± 2.13 N/kg, surgical = 4.75 ± 2.18 N/kg, *p* = 0.036) forces on the healthy side were significantly larger than the surgical side at the peak moment of tibial compression forces during jogging (Table 2; Figure 3). There was no difference in other bilateral muscle forces at the peak moment of tibial compression forces during jogging and walking (Tables 2–4 and Figures 3, 4).

**TABLE 2:** Muscle forces at the peak moment of tibial compression forces on the both sides during jogging.

Muscle name	Healthy side (N/kg)	Surgical side (N/kg)	*p*-value
rectus femoris	32.08 ± 10.79	22.58 ± 9.16^*^	**0.035**
vastus intermedius	6.56 ± 2.35	5.41 ± 2.29	0.122
vastus medialis	6.08 ± 2.13	4.75 ± 2.18^*^	**0.036**
vastus lateralis	9.10 ± 3.44	7.66 ± 3.29	0.073
long head of biceps femoris	1.53 ± 1.28	1.15 ± 0.46	0.390
semimembranosus	1.17 ± 0.73	0.96 ± 0.70	0.378
semitendinosus	0.63 ± 0.28	0.53 ± 0.25	0.395
short head of biceps femoris	1.53 ± 0.49	1.07 ± 0.52	0.095
lateral head of the gastrocnemius	2.73 ± 1.70	3.23 ± 0.95	0.453
medial head of gastrocnemius	8.22 ± 3.27	7.93 ± 1.15	0.775
gracilis	2.08 ± 1.22	1.60 ± 1.26	0.383
sartorius	0.13 ± 0.18	0.11 ± 0.10	0.676

*Significant difference between groups (*p* ≤ 0.05).

The bold values were used to highlight the *p*-values ≤ 0.05.

**TABLE 3:** Muscle forces at the first peak moment of tibial compression forces on the both sides during walking.

Muscle name	Healthy side (N/kg)	Surgical side (N/kg)	*p*-value
rectus femoris	10.35 ± 3.98	8.17 ± 5.75	0.129
vastus intermedius	2.31 ± 1.33	1.76 ± 1.29	0.115
vastus medialis	2.00 ± 1.30	1.60 ± 1.45	0.198
vastus lateralis	3.26 ± 1.60	2.77 ± 2.12	0.215
long head of biceps femoris	1.44 ± 0.75	1.33 ± 0.92	0.814
semimembranosus	2.13 ± 0.99	2.34 ± 1.63	0.791
semitendinosus	0.74 ± 0.49	0.69 ± 0.53	0.855
short head of biceps femoris	2.18 ± 0.96	1.60 ± 0.74	0.171
lateral head of the gastrocnemius	1.89 ± 1.27	1.60 ± 1.73	0.534
medial head of gastrocnemius	6.58 ± 6.66	3.13 ± 2.61	0.221
gracilis	1.45 ± 1.46	0.81 ± 1.08	0.096
sartorius	0.16 ± 0.29	0.05 ± 0.05	0.275

**TABLE 4:** Muscle forces at the second peak moment of tibial compression forces on the both sides during walking.

Muscle name	Healthy side (N/kg)	Surgical side (N/kg)	*p*-value
rectus femoris	3.28 ± 2.02	5.00 ± 4.15	0.106
vastus intermedius	0.13 ± 0.12	0.23 ± 0.29	0.106
vastus medialis	0.11 ± 0.12	0.48 ± 0.90	0.138
vastus lateralis	0.16 ± 0.12	0.21 ± 0.22	0.130
long head of biceps femoris	0.33 ± 0.36	0.30 ± 0.46	0.630
semimembranosus	0.43 ± 0.38	0.51 ± 0.95	0.722
semitendinosus	0.22 ± 0.13	0.25 ± 0.41	0.765
short head of biceps femoris	0.88 ± 0.60	0.77 ± 0.73	0.560
lateral head of the gastrocnemius	3.19 ± 2.02	5.08 ± 4.36	0.302
medial head of gastrocnemius	9.88 ± 6.47	7.06 ± 4.39	0.187
gracilis	1.26 ± 1.10	1.24 ± 1.63	0.966
sartorius	0.02 ± 0.01	0.01 ± 0.02	0.635

There was no difference in bilateral CCI_flex-ext_ at the peak moment of tibial compression forces during jogging and walking (Table 5).

**TABLE 5:** CCI_flex-ext_ at peak moments of tibial compression forces on the both sides during jogging and walking.

	Healthy side	Surgical side	*p*-value
Peak in jogging	−0.46 ± 0.32	0.30 ± 0.32	0.062
First peak in walking	0.24 ± 0.22	0.28 ± 0.19	0.296
Second peak in walking	0.86 ± 0.11	0.62 ± 0.40	0.051

Participants’ knee flexion angles (healthy = 49.50 ± 3.77°, surgical = 44.51 ± 7.03°, *p* = 0.042) and ankle dorsiflexion angles (healthy = 23.84 ± 6.50°, surgical = 19.88 ± 5.04°, *p* = 0.046) on the healthy side were significantly higher than that on the surgical side at the peak moment of tibial compression forces during jogging (Table 6). Participants’ ankle dorsiflexion angles (healthy = 6.94 ± 3.85°, surgical = 4.91 ± 4.09°, *p* = 0.038) on the healthy side were significantly higher than that on the surgical side at the first peak moment of tibial compression forces during walking (Table 6). There was no difference in other bilateral joint angles at the peak moment of tibial compression forces during jogging and walking (Table 6).

**TABLE 6:** Joint angles at peak moments of tibial compression forces on the both sides during jogging and walking.

	Peak in jogging			First peak in walking			Second peak in walking		
	Healthy side	Surgical side	*p*-value	Healthy side	Surgical side	*p*-value	Healthy side	Surgical side	*p*-value
ankle dorsiflexion (°)	23.84 ± 6.50	19.88 ± 5.04^*^	**0.046**	6.94 ± 3.85	4.91 ± 4.09^*^	**0.038**	19.41 ± 6.00	18.60 ± 3.96	0.574
knee flexion (°)	49.50 ± 3.77	44.51 ± 7.03^*^	**0.042**	30.12 ± 3.22	28.73 ± 4.87	0.241	11.89 ± 4.33	14.13 ± 4.16	0.089
hip flexion (°)	42.48 ± 11.49	41.56 ± 8.63	0.352	37.59 ± 9.95	40.84 ± 9.17	0.092	−4.41 ± 4.84	3.05 ± 6.59	0.573
hip adduction (°)	5.70 ± 3.41	5.36 ± 5.63	0.445	2.65 ± 5.06	0.34 ± 4.77	0.178	10.56 ± 1.88	8.60 ± 5.01	0.223
hip rotation (°)	7.17 ± 2.05	5.40 ± 3.02	0.120	12.46 ± 3.83	11.22 ± 4.14	0.327	5.92 ± 3.74	5.99 ± 6.06	0.951

*Significant difference between groups (*p* ≤ 0.05).

The bold values were used to highlight the *p*-values ≤ 0.05.

## 4 Discussion

The purpose of this study was to compare the difference in bilateral tibial contact force for unilateral ACLR patients in order to assess the risk of knee osteoarthritis. The EMG-assisted method was used to predict muscle forces based on the neuromusculoskeletal model. We evaluated model simulation results in two ways. Firstly, the joint net moment calculated by EMG-assisted methods was compared to the ID joint net moment. Studies that use the EMG-driven method usually evaluated results in this way (Lloyd and Besier, 2003; Shao et al., 2009; Nikooyan et al., 2012; Sartori et al., 2012; Sartori et al., 2014). Our results showed a high correlation and low error between these two results. Secondly, the tibial compression force calculated by the model was compared with the measured values of knee implants obtained by the patients after total knee replacement when they performed similar movement tasks. Our results showed a similar trend and magnitude to Bergmann et al.’s results (Bergmann et al., 2014). Our results in jogging were larger than the Bergmann et al.’s results. This may be due to differences in the participants’ age, jogging speed, and history of joint surgery.

The results of tibial compression forces support our first hypothesis that the tibial compression force on the surgical side for ACLR patients will be smaller than on the healthy side. This study showed that the peak tibial compression force on the healthy side was higher than on the surgical side during jogging. This result is consistent with the literature. One study followed a group of ACLR patients for up to 5 years after surgery and reported that those who developed osteoarthritis 5 years later had lower tibial compression forces in their gait 6 months to 2 years after surgery than those who did not (Wellsandt et al., 2016). Another study suggested that knee joints with ACLR surgery showed lower tibial contact forces during jogging, walking, and side stepping than the healthy group (Saxby et al., 2016).

Lower tibial compression force may lead to an increased risk of knee osteoarthritis. On the one hand, the thickness of healthy cartilage increases under a higher repetitive load and joint unloading is associated with regional cartilage thinning after ACLR (Koo and Andriacchi, 2007). A smaller tibial compression force may cause regional cartilage thinning and joint degeneration. The patient’s physical activity level will gradually increase to return to daily life after surgery. If the joint load-bearing capacity has not recovered to its pre-operative level, the risk of knee osteoarthritis may be increased. On the other hand, Saxby et al. suggested that the total area of contact between the tibiofemoral articulating surfaces may reduce because of a smaller knee flexion angle and knee flexion excursion, so articular contact forces were focused to smaller regions although the magnitudes of tibial contact forces were small (Saxby et al., 2016). Our result also supported that the surgical side had a smaller knee flexion angle at the moment of peak tibial compression force than the healthy side. This may be another explanation for the increased risk of knee osteoarthritis following ACLR.

The results of biomechanical characteristics support our second and third hypothesis that there will be differences in muscle forces around the knee joint and lower limb kinematics between the surgical and healthy sides during walking and jogging. The reason for the lower tibial compression force after ACLR may be the lower quadriceps muscle forces. We analyzed the lower extremity biomechanical characteristics at the moment of peak tibial compression force to understand why the peak tibial compression force on the surgical side is smaller than that on the healthy side. In this paper, it was suggested that compared with the healthy side, the ACLR side had a smaller knee flexion and ankle dorsiflexion angle at the peak moment of tibial compression force, which was consistent with the results of multiple previous studies (Lewek et al., 2002; Tsai et al., 2012; Kaur et al., 2016; Saxby et al., 2016). The reduced knee flexion angle is a typical change of gait after ACLR. However, this change does not appear to be the cause of the decreased tibial compression force since a smaller knee flexion and ankle dorsiflexion angle should theoretically increase the tibial compression force due to the direction of the force of gravity. Therefore, we infer that changes in knee and ankle joint motion patterns are not the primary reason for the decreased tibial compression force. Instead, muscle forces around the knee are directly related to the joint load (Saxby et al., 2016), and we found that the quadriceps femoris force on the surgical side was lower than that on the healthy side, which may be responsible for the reduced tibial compression force. Interestingly, we did not find any differences in CCI_flex-ext_ between the two sides at the peak moment of tibial compression force. The activation patterns of muscles can affect joint contact forces, and that the high degree of co-contraction of muscles may increase joint compression forces. This inference is based on the positive correlation between muscle activation levels and muscle strength. However, although this study reported differences in bilateral tibial compression forces and muscle forces, it did not show any differences in CCI_flex-ext_. One possible explanation is that the muscle model used in this study is based on the Hill model, which mainly includes the relationship between muscle force and muscle length, velocity, and activation level (Sartori et al., 2014; Pizzolato et al., 2015). CCI_flex-ext_ only reflects the characteristics of muscle activation, but this is not the only factor that affects the muscle force.

The neuromusculoskeletal model for the EMG-assisted method in this paper only included knee flexion-extension degrees of freedom. Although the range of motion of the knee joint in jogging and walking is tiny, the influence of muscles around other joints on the development of knee osteoarthritis can be further analyzed in the future study. In addition, the sample size of this study was not large, and the participants’ postoperative time ranged from 1.5 to 3.5 years, which does not fully explain the time frame for the increased risk of knee osteoarthritis, but additional participant data is being collected to test the approach across a larger cohort.

## 5 Conclusion

Unilateral ACLR patients had smaller tibial compression forces on the surgical side than on the healthy side while jogging, which may increase the risk of knee osteoarthritis. The main reason may be that the quadriceps muscles do not exert enough force. We recommend that ACLR patients pay attention to strengthening their quadriceps muscle through strength training during the postoperative rehabilitation process, and try to increase the use of the quadriceps muscle on the surgical side to an appropriate level in daily activities.

## Data Availability

The raw data supporting the conclusion of this article will be made available by the authors, without undue reservation.
