# Supplementary material for: Evaluating the risk of knee osteoarthritis following unilateral ACL reconstruction based on an EMG-assisted method
Source: Front Physiol. 2023 Apr 21;14:1160261. doi: 10.3389/fphys.2023.1160261 (PMC10160379; doi:10.3389/fphys.2023.1160261)
Supplement: Supplementary file 1 [file DataSheet1.docx]

Supplementary Material

Evaluating the risk of knee osteoarthritis after unilateral ACL reconstruction based on neuromusculoskeletal model

Ting Long^1^, Justin Fernandez^1,2^, Hui Liu^3^, Hanjun Li^4*^

*** Correspondence:** Hanjun Li: lihanjun@bsu.edu.cn

# Supplementary Figures and Tables

## Supplementary Figures


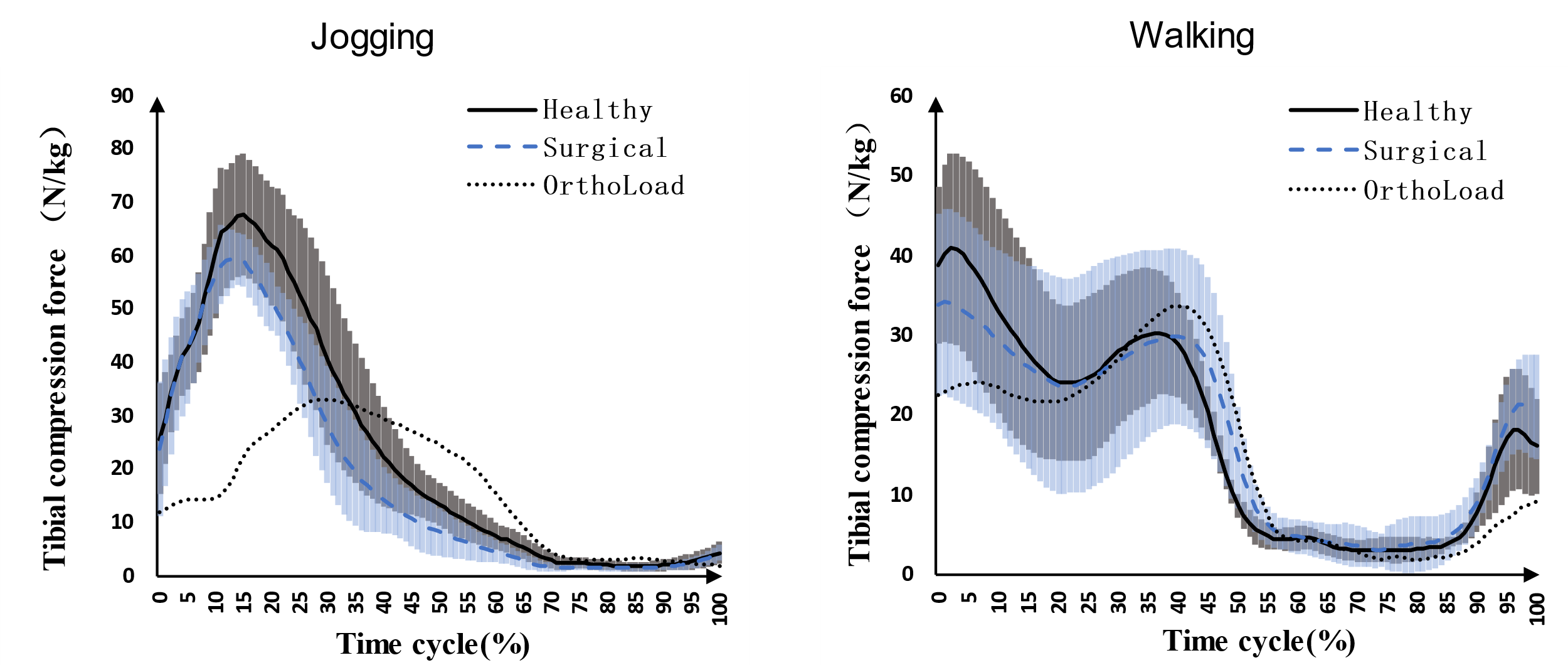


**Supplementary Figure 1.** Knee compression forces on the both sides in this study and OrthoLoad dataset during jogging and walking

## Supplementary Tables

**Supplementary Table 1.** The setting of model scaling

| Segment | Scaling marker pair |
| --- | --- |
| torso | bilateral acromium; vertex of the head and midpoint of the fourth and fifth lumbar vertebrae |
| pelvis | bilateral anterior superior iliac spines |
| femur | anterior superior iliac spines and lateral femoral condyles |
| tibia | lateral femoral condyles and lateral fibular condyles |
| talus | heels and toe tips |
| calcaneus | heels and toe tips |
| toes | heels and toe tips |

**Supplementary Table 2.** The setting of muscle excitations mapping (There was no muscle excitations mapping in gracilis and sartorius, they were generated by optimization process)

| MTU | Experimental excitation |
| --- | --- |
| rectus femoris | rectus femoris |
| vastus intermedius | 0.5vastus medialis+0.5vastus lateralis |
| vastus medialis | vastus medialis |
| vastus lateralis | vastus lateralis |
| long head of biceps femoris | long head of biceps femoris |
| short head of biceps femoris |  |
| semitendinosus | semitendinosus |
| semimembranosus |  |
| lateral head of the gastrocnemius | lateral head of the gastrocnemius |
| medial head of gastrocnemius | medial head of gastrocnemius |
| gracilis | / |
| sartorius | / |
